# Supplementary material for: Resolving the full spectrum of human genome variation using Linked-Reads
Source: Genome Res. 2019 Apr;29(4):635–45. doi: 10.1101/gr.234443.118 (PMC6442396; doi:10.1101/gr.234443.118)
Supplement: Supplemental Material [file supp_gr.234443.118_Supplemental_longranger-2.2.zip › longranger-2.2.tar.bz2!/lr2.2-public/lib/stan/cmdstan/stan/doc/api/html/vector__vari_8hpp_source.html]

Stan: src/stan/agrad/rev/internal/vector\_vari.hpp Source File


|  |  |
| --- | --- |
|  | Stan  2.5.0  probability, sampling & optimization |


- Main Page
- Related Pages
- Namespaces
- Classes
- Files

- File List
- File Members

All Classes Namespaces Files Functions Variables Typedefs Enumerations Enumerator Friends Macros Pages

- src
- stan
- agrad
- rev
- internal

vector\_vari.hpp

Go to the documentation of this file.

1 #ifndef STAN\_\_AGRAD\_\_REV\_\_INTERNAL\_\_VECTOR\_VARI\_HPP

2 #define STAN\_\_AGRAD\_\_REV\_\_INTERNAL\_\_VECTOR\_VARI\_HPP

3

4 #include <vector>

5 #include <stan/agrad/rev/var.hpp>

6 #include <stan/agrad/rev/vari.hpp>

7

8 namespace stan {

9  namespace agrad {

10

11  class op\_vector\_vari : public vari {

12  protected:

13  const size\_t size\_;

14  vari\*\* vis\_;

15  public:

16  op\_vector\_vari(double f, const std::vector<stan::agrad::var>& vs) :

17  vari(f),

18  size\_(vs.size()) {

19  vis\_ = (vari\*\*) operator new(sizeof(vari\*) \* vs.size());

20  for (size\_t i = 0; i < vs.size(); ++i)

21  vis\_[i] = vs[i].vi\_;

22  }

23  vari\* operator[](size\_t n) const {

24  return vis\_[n];

25  }

26  size\_t size() {

27  return size\_;

28  }

29  };

30

31  }

32 }

33 #endif

stan::agrad::op\_vector\_vari::op\_vector\_vari

op\_vector\_vari(double f, const std::vector< stan::agrad::var > &vs)

**Definition:** vector\_vari.hpp:16

vari.hpp

stan::agrad::op\_vector\_vari

**Definition:** vector\_vari.hpp:11

stan::agrad::op\_vector\_vari::size\_

const size\_t size\_

**Definition:** vector\_vari.hpp:13

stan::agrad::vari

The variable implementation base class.

**Definition:** vari.hpp:28

stan::agrad::op\_vector\_vari::vis\_

vari \*\* vis\_

**Definition:** vector\_vari.hpp:14

var.hpp

stan::agrad::op\_vector\_vari::operator[]

vari \* operator[](size\_t n) const

**Definition:** vector\_vari.hpp:23

stan::agrad::op\_vector\_vari::size

size\_t size()

**Definition:** vector\_vari.hpp:26


---

[ Stan Home Page ]

*© 2011–2014,
Stan Development Team.*
